# Pyridoxine supplementation corrects vitamin B6 deficiency but does not improve inflammation in patients with rheumatoid arthritis

**DOI:** 10.1186/ar1839

**Published:** 2005-10-14

**Authors:** En-Pei I Chiang, Jacob Selhub, Pamela J Bagley, Gerard Dallal, Ronenn Roubenoff

**Affiliations:** 1 Department of Food Science and Biotechnology, National Chung-Hsing University, 250 Kuo-Kuang Road, Taichung, Taiwan 402, Republic of China; 2 Vitamin Metabolism Laboratory, Jean Mayer USDA Human Nutrition Research Center on Aging, Tufts University, 711 Washington Street, Boston, MA 02111, USA; 3 Biostatistics Unit, Jean Mayer USDA Human Nutrition Research Center on Aging, Tufts University, 711 Washington Street, Boston, MA 02111, USA; 4 Nutrition, Exercise Physiology, and Sarcopenia Laboratory Jean Mayer USDA Human Nutrition Research Center on Aging, Tufts University, 711 Washington Street, Boston, MA 02111, USA; 5 Tufts-New England Medical Center, 136 Harrison Avenue, Boston, MA 02111, USA

## Abstract

Patients with rheumatoid arthritis have subnormal vitamin B6 status, both quantitatively and functionally. Abnormal vitamin B6 status in rheumatoid arthritis has been associated with spontaneous tumor necrosis factor (TNF)-α production and markers of inflammation, including C-reactive protein and erythrocyte sedimentation rate. Impaired vitamin B6 status could be a result of inflammation, and these patients may have higher demand for vitamin B6. The aim of this study was to determine if daily supplementation with 50 mg of pyridoxine for 30 days can correct the static and/or the functional abnormalities of vitamin B6 status seen in patients with rheumatoid arthritis, and further investigate if pyridoxine supplementation has any effects on the pro-inflammatory cytokine TNF-α or IL-6 production of arthritis. This was a double-blinded, placebo-controlled study involving patients with rheumatoid arthritis with plasma pyridoxal 5'-phosphate below the 25th percentile of the Framingham Heart Cohort Study. Vitamin B6 status was assessed via plasma and erythrocyte pyridoxal 5'-phosphate concentrations, the erythrocyte aspartate aminotransferase activity coefficient (αEAST), net homocysteine increase in response to a methionine load test (ΔtHcy), and 24 h urinary xanthurenic acid (XA) excretion in response to a tryptophan load test. Urinary 4-pyridoxic acid (4-PA) was measured to examine the impact of pyridoxine treatment on vitamin B6 excretion in these patients. Pro-inflammatory cytokine (TNF-α and IL-6) production, C-reactive protein levels and the erythrocyte sedimentation rate before and after supplementation were also examined. Pyridoxine supplementation significantly improved plasma and erythrocyte pyridoxal 5'-phosphate concentrations, erythrocyte αEAST, urinary 4-PA, and XA excretion. These improvements were apparent regardless of baseline B6 levels. Pyridoxine supplementation also showed a trend (*p* < 0.09) towards a reduction in post-methionine load ΔtHcy. Supplementation did not affect pro-inflammatory cytokine production. Although pyridoxine supplementation did not suppress pro-inflammatory cytokine production in patients with rheumatoid arthritis, the suboptimal vitamin B6 status seen in rheumatoid arthritis can be corrected by 50 mg pyridoxine supplementation for 30 days. Data from the present study suggest that patients with rheumatoid arthritis may have higher requirements for vitamin B6 than those in a normal healthy population.

## Introduction

Patients with rheumatoid arthritis have reduced circulating levels of vitamin B6 compared to healthy subjects [1-3]. We have demonstrated that low plasma pyridoxal 5'-phosphate levels reflect the impaired functional vitamin B6 status in these patients. Plasma pyridoxal 5'-phosphate levels correlated with both the net homocysteine increase in response to a methionine load test (ΔtHcy) and 24 h urinary xanthurenic acid excretion in response to a tryptophan load test (XA) [4]. We also demonstrated that the inadequate vitamin B6 status seen in patients with rheumatoid arthritis was not due to insufficient dietary intake or excessive excretion, but was related to the inflammatory status of their underlying disease [4,5]. Abnormal vitamin B6 status in rheumatoid arthritis has been associated with spontaneous tumor necrosis factor (TNF)-α production [1] and markers of inflammation, including C-reactive protein (CRP) and erythrocyte sedimentation rate [5]. We recently showed that adjuvant arthritis caused tissue-specific depletion of vitamin B6 in rats [6], suggesting that the impaired vitamin B6 metabolism in patients with rheumatoid arthritis result from inflammation, and these patients may have higher requirements for vitamin B6 than those in a normal healthy population.

Vitamin B6 supplementation for patients with rheumatoid arthritis has been considered. Earlier studies reported that short-term pyridoxine treatment normalized tryptophan metabolism in patients with rheumatoid arthritis, but did not improve arthritis symptoms [7-9]. These studies were limited by small sample size, absence of placebo controls or blinding, and limited assessment of B6 metabolism, relying instead on pyridoxal 5'-phosphate levels, which are altered by inflammation itself. Furthermore, the cause of subnormal vitamin B6 status in rheumatoid arthritis remains to be determined and it is not known whether vitamin B6 supplementation improves functional vitamin B6 indices in these patients. The present study is the first one to systematically investigate the efficacy of vitamin B6 supplementation on static and functional vitamin B6 indices in patients with rheumatoid arthritis.

Although vitamin B6 supplementation appeared ineffective for symptom relief in rheumatoid arthritis, it should still be considered in these patients because of the potential adverse consequences of vitamin B6 insufficiency. Vitamin B6 deficiency in animals has been related to atherosclerotic lesions [10]. More recently, researchers demonstrated a relationship between vitamin B6 deficiency and atherosclerosis in human population-based studies, and they reported that this relationship was independent of plasma total homocysteine (tHcy) levels both before and after methionine loading [11,12]. Furthermore, vitamin B6 deficiency is associated with post-methionine load hyperhomocysteinemia, another known independent risk factor for cardiovascular disease [13-15]. We previously reported that patients with rheumatoid arthritis have mild but significantly elevated ΔtHcy in response to methionine load compared to age- and gender-matched healthy controls [2,16]. This led us to evaluate the efficacy of giving vitamin B6 supplements to rheumatoid arthritis patients with respect to decreasing the elevated ΔtHcy and improve functional vitamin B6 status. The goal of the present study was to investigate whether treatment with 50 mg pyridoxine for 30 days improves static and functional indices of vitamin B6 status in patients with rheumatoid arthritis.

## Materials and methods

### Study population

Thirty six adults with rheumatoid arthritis were recruited through the Tufts New England Medical Center (NEMC) Rheumatology Clinic as previously described [5]. Written informed consent was obtained from all subjects in accordance with the regulations of the NEMC/Tufts University Human Investigation Review Committee. Briefly, men and women over 18 years old fulfilling the American College of Rheumatology criteria for rheumatoid arthritis were eligible [17]. Patients with pregnancy, oral contraceptive use, anemia (hemoglobin ≤ 10 mg/dl), thrombocytopenia (platelet count ≤ 50,000/ul), abnormal liver transaminase (serum aspartate aminotransferase or alanine aminotransferase ≥ 50 IU/l), renal insufficiency (serum creatinine ≥ 1.5 mg/dl), diabetes, or cancer were excluded. Patients taking supplements containing vitamin B6 were asked to stop for ≥ 1 month before their participation in the study.

### Study protocol

This double-blinded, randomized and placebo controlled trial was conducted in the General Clinical Research Center (GCRC) at Tufts-NEMC. Prior to enrollment, blood screening and urinalysis were performed to ensure qualification and to identify individuals with low circulating vitamin B6 for the study. To test the efficacy of vitamin B6 supplementation on those patients with reduced plasma pyridoxal 5'-phosphate, baseline (phase 1) vitamin B6 status was determined using a two day test procedure as follows. Patients taking methotrexate were asked to come at least 24 h after their weekly dose of this drug. On the first day of the evaluation (day 1), each subject arrived in the GCRC at 8 a.m. after having eaten breakfast. Each subject received a standard oral tryptophan load test (5 g powdered L-tryptophan dissolved in chocolate milk; Ajinomoto, Teaneck, NJ, USA) and collected urine for the next 24 h. The urine was kept refrigerated without additives during the collection period. Separate 24 h urine collection was done in the week prior to day 1 for the measurement of baseline XA and 4-pyridoxic acid (4-PA) excretion.

Subjects were asked to fast overnight starting at 8 p.m. on day 1 for the methionine load test next morning. After completion of the 24 h urine collection in the morning of day 2, each subject received a standard methionine load test [18]. Baseline fasting blood was drawn in a tube containing ethylenediaminetetraacetic acid (EDTA) (Becton Dickinson, Franklin Lakes, NJ, USA) for determination of plasma pyridoxal 5'-phosphate, fasting tHcy level, erythrocyte pyridoxal 5'-phosphate concentration, erythrocyte aspartate aminotransferase activity (EAST), and CRP concentrations. Aliquots were also collected for routine hematology and chemistry analyses. Peripheral blood mononuclear cells (PBMC) were collected from heparinized blood and isolated by Ficoll-Hypaque centrifugation, then washed and cultured for 24 h in 96-well flat-bottom plates with ultrafiltered, pyrogen-free RPMI 1640 medium (Sigma, St. Louis, MO, USA) that was supplemented with 100 μg/ml streptomycin and 100 U/ml penicillin, with 1% autologous heat-inactivated pooled serum and 1% L-glutamine. After incubation, plates were then frozen at -80°C until assay.

After collection of fasting blood on day 2, each patient was then given a standard oral methionine load test (100 mg/kg body weight powdered methionine dissolved in orange juice; Ajinomoto, Teaneck, NJ, USA). Blood was drawn 4 h after the methionine load for determination of the post-load tHcy level. Fasting plasma pyridoxal 5'-phosphate levels were determined within 1 week and the level was compared to the 25th percentile of the Framingham Offspring Heart Cohort [19]. Patients with a plasma pyridoxal 5'-phosphate level within the lowest quartile of the appropriate age and gender Framingham population (cycle 6, offspring group) were recruited for the supplementation phase of the study (phase 2). The 25^th ^percentile cutoff for plasma pyridoxal 5'-phosphate in women below 55 years and women at or above 55 years were 33.7 and 37.5 nmol/l, respectively. For men below 55 years and for men at or above 55 years it was 49.7 and 35.6 nmol/l, respectively [19].

### Study interventions

Qualified subjects started taking the study treatment within one week of plasma pyridoxal 5'-phosphate analysis. These subjects were randomly assigned through the NEMC pharmacy to receive either active vitamin B6 (B6 group) or placebo (placebo group) tablets in double-blinded fashion for 30 days. To minimize the potential confounding effect of methotrexate and prednisone treatment on the functional tests, subjects were stratified by prednisone and methotrexate treatment, and then the subjects in each group were randomized to receive either active or placebo treatment. The randomization procedure was under guidance of a statistician and performed by registered pharmacists not directly involved in the present study.

The placebo tablet, made specifically for the study, was identical in appearance as well as ingredients to the active tablet, except that the active tablet (Nutro Laboratories, South Plainfield, NJ, USA) contained 50 mg of pyridoxine hydrochloride and the placebo did not (Tishcon Corp., Westbury, NY, USA). Both tablets contained microcrystalline cellulose, croscarmellose sodium, calcium phosphate, stearic acid, and magnesium stearate, ingredients commonly found in over-the-counter vitamin B6 supplements. Each phase 2 participant was asked to take one assigned tablet daily throughout the 30 day period. To assure compliance with the treatment regimen, each subject was given a personal study calendar with the 30 supplement days highlighted. The subject was asked to record the time of ingestion of each tablet on the calendar. In addition, the study coordinator made phone calls to remind each subject to take the tablets during the 30 day supplement period. The subjects were asked to return the bottle for a tablet count at the end of the 30 day treatment. To test the efficacy of the vitamin B6 supplementation, each subject went through the same 2 day testing procedure described above at the end of the 30 day supplementation period.

### Laboratory analyses

Blood hematology and chemistry analyses and urinalysis were performed at the Clinical Laboratory of NEMC, Boston, MA. CRP concentrations were determined by enzyme immunoassay kit (Virgo CRP150 kit, Hemagen, Waltham, MA, USA). Pyridoxal 5'-phosphate concentration was assayed by the tyrosine decarboxylase enzymatic procedure of Camp *et al*. [20] with a modification of the extraction procedure for plasma and erythrocytes. The modification is described as follows: a 20 μl plasma aliquot was precipitated with 4 volumes of 5% trichloroacetic acid for deproteinization. Erythrocytes were washed with 0.9% saline 3 times and the freshly washed erythrocytes were extracted with an equal volume of 10% (w/v) perchloroacetic acid. After centrifugation, the supernatants were stored at -70°C until the analysis. The erythrocyte pyridoxal 5'-phosphate results were expressed as nmol/l of packed erythrocyte at a hemotocrit of 100%. Fasting and post-methionine load plasma tHcy concentrations [21] and 4-PA [22] were determined by high performance liquid chromatography (HPLC) using a Hitachi L-7100 intelligent pump connected to an L-7400 UV detector (Hitachi, Tokyo, Japan). Baseline and post-tryptophan load urinary XA were measured by a colorimetric method [23]. EAST activity was measured using the Cobas Fara II Centrifugal Analyzer (Roche Dianostic system Inc., Nutley, NJ, USA) [24]. The ratio of pyridoxal 5'-phosphate saturated and unsaturated enzyme is expressed as the activity coefficient αEAST. Plasma TNF-α concentrations and PBMC TNF-α and IL-6 production was assayed with the commercially available quantitative enzyme immunoassays (Quantikine, R&D Systems, Minneapolis, MN, USA). Total PBMC cytokine production was measured in unstimulated cells (spontaneous production).

### Statistical analysis

Differences in means between the baseline indices of the active group versus the placebo group were evaluated by Student's t-tests to examine if the randomization was successful. Differences were considered significant if the two-tailed p-value was <0.05. Plasma pyridoxal 5'-phosphate, tHcy, urinary XA, and 4-PA levels were log-transformed to achieve normality. Analysis of covariance (ANCOVA) was used to test the treatment effect of pyridoxine. The model was adjusted for the baseline (phase 1) value. A Pearson's correlation coefficient was calculated to examine the relationship between plasma pyridoxal 5'-phosphate levels and the inflammatory marker CRP before and after the treatment period. All statistical analyses were performed using Systat 10.0 for Windows ™ (SPSS, Chicago, IL, USA).

## Results

Thirty-six patients with rheumatoid arthritis who met the eligibility requirements for the study were recruited for phase 1 of the study. Three patients dropped out because of scheduling problems or due to the concern over ingestion of the methionine and/or tryptophan. Of the 33 patients who completed the phase 1 procedure, 28 patients (85%) were found to have plasma pyridoxal 5'-phosphate levels within the lowest quartile of the age- and gender-matched population of the Framingham Offspring Study and thus qualified for the supplementation phase (Table 1). The number of pills consumed by each participant during the treatment period was divided by the total number of pills supplied to each subject (n = 30). The average percentage of pill consumption and the standard deviation in each group was calculated. Based on the tablet counts after the completion of the study, the compliance of treatment regimen was 97.8 ± 6.3 (%) for the B6 group and 98.3 ± 5.2 (%) for the placebo group. Baseline characteristics in the B6 and the placebo groups were comparable, indicating that randomization was appropriate (Table 1).

Indicators of vitamin B6 status before and after treatment are shown in Table 2. All markers of vitamin B6 status improved significantly in the B6 group after supplementation, except for net increase in total homocysteine concentration, which only showed a modest trend towards improvement. None of the vitamin B6 status parameters showed significant improvements after treatment in the placebo group. Analysis of co-variance further demonstrated that initial levels of plasma pyridoxal 5'-phosphate, ΔtHcy, post-load urinary XA, αEAST (Table 2) and CRP and erythrocyte sedimentation rate (ESR) (Table 3) in phase 1 were strong predictors of those indicators after treatment in phase 2. After adjusting for the initial levels in (before treatment), the vitamin B6 supplementation significantly improved plasma and erythrocyte pyridoxal 5'-phosphate concentrations, αEAST, post-load XA and 24 h 4-PA excretion. We found a trend for normalization of plasma ΔtHcy in the vitamin B6 treatment versus placebo group (*p* = 0.086, ANCOVA). In patients who had abnormal ΔtHcy (above 15 μmol/l) before treatment (n = 22/28), the effect of vitamin B6 treatment was significant (*p* < 0.02). Plasma pyridoxal 5'-phosphate and ΔtHcy levels were related to CRP in patients with rheumatoid arthritis [5], thus CRP could be a potential targets for vitamin B6 supplementation. The correlations between CRP and plasma pyridoxal 5'-phosphate and ΔtHcy disappeared in the B6 group after supplementation, whereas the relationships remained in the placebo group after the 30 day treatment (CRP versus plasma pyridoxal 5'-phosphate, r = -0.61, *p* = 0.02; CRP versus ΔtHcy, r = 0.48, *p* = 0.098) (n = 14). We found that vitamin B6 supplementation had no effect on inflammatory cytokines, plasma CRP, ESR, or rheumatoid factor levels in these patients (Table 3).

## Discussion

Abnormal vitamin B6 metabolism has been reported in rheumatoid arthritis for decades [3,7,8,25,26]. Considering the close associations between vitamin B6 indices and the clinical and biochemical inflammatory markers [5], it is likely that inflammation causes vitamin B6 deficiency, yet it is also possible that impaired vitamin B6 status contributes to more severe inflammation in these patients. The present study demonstrates that 50 mg of pyridoxine hydrochloride supplementation for 1 month can significantly improve vitamin B6 status in patients with rheumatoid arthritis regardless of the etiology of such inadequacy. In contrast, vitamin B6 supplementation was ineffective in suppressing inflammatory cytokine production, or reducing ESR, plasma CRP or rheumatoid factor levels in these patients. As improving vitamin B6 status did not alleviate inflammation, it is unlikely that vitamin B6 inadequacy directly causes or worsens the inflammatory condition. We suggest that the impaired vitamin B6 metabolism in patients with rheumatoid arthritis results from inflammation, and these patients may be in higher demand of vitamin B6 to cope with the ongoing inflammatory condition. In the present study, 85% (28/33) of our participants had a plasma pyridoxal 5'-phosphate concentration below the 25^th ^percentile of the Framingham population data at baseline. We previously reported the presence of functional vitamin B6 inadequacy in rheumatoid arthritis patients: ΔtHcy after a methionine load test was significantly higher in patients with rheumatoid arthritis than healthy matched controls, indicating that impaired trans-sulfuration accompanied the low plasma vitamin B6 levels [2,16].

Both clinical studies and animal experiments suggest that inflammation causes tissue specific depletion of vitamin B6 [4,5,16]. It is not clear how different tissues may respond to vitamin B6 supplementation during inflammation. While patients with rheumatoid arthritis have abnormal systemic functional status of vitamin B6 (as measured by ΔtHcy level in response to a methionine load test), they appear to have normal functional vitamin B6 status specifically in the erythrocytes (as measured by αEAST) [16]. Previously, we demonstrated that vitamin B6 status in erythrocytes is more sensitive to dietary vitamin B6 intake compared to plasma pyridoxal 5'-phosphate concentration or functional indices, including ΔtHcy and XA excretion in patients with rheumatoid arthritis [4]. Based on this observation, we expected αEAST to be more responsive to vitamin B6 supplementation compared to the methionine load test. The results from the present study support our speculation. All subjects in the B6 group had improvements in αEAST, including those patients who had a normal initial αEAST before supplementation. After supplementation, the mean reduction in αEAST was 32% of the original αEAST, and all individuals after the supplementation had an αEAST in the desirable range (αEAST ≤ 1.5) suggested by Leklem [27]. Individuals in the placebo group had no significant change in αEAST. In conclusion, erythrocyte αEAST reflects vitamin B6 intake rather than systemic B6 functional status, and is more sensitive to vitamin B6 supplementation in these patients.

Post-tryptophan load XA excretion above 146.2 μmol/day (30 mg/day) was considered as the cutoff for inadequacy in healthy volunteers after ingestion of 5 g of L-tryptophan [28]. In our phase 1 screening, 19 of the 28 patients had post-tryptophan XA excretion levels above this threshold. After 30 days of vitamin B6 treatment, 13 of the 14 patients in the B6 treated group had normal levels of post-tryptophan load XA excretion, whereas only 2 of the patients with abnormal XA in the placebo group fell in the 'adequate range' after treatment. Our results suggest that pyridoxine treatment can normalize tryptophan metabolism in those patients with abnormal tryptophan metabolism.

With respect to different indicators for functional vitamin B6 status, we found that the effect of the pyridoxine treatment on the response to a methionine load test was not as strong as αEAST or post-load XA. There was a mild vitamin B6 treatment effect after adjusting for initial ΔtHcy in phase 1 (ANCOVA, *p* = 0.086). Twenty-five percent (7/28) of these patients had a 'normal' ΔtHcy (below 15 μmol/l) before treatment, which might account for the overall modest treatment effect of pyridoxine in our study.

Subnormal vitamin B6 status has also been shown in some asthma patients. It was reported that vitamin B6 supplementation (20 mg/day) for 6 weeks significantly reduced post-methionine load ΔtHcy in asthma patients with low vitamin B6 status, but it had no significant effect in controls with normal ΔtHcy response [29]. The initial ΔtHcy level in our present study is comparable with those in the above study (pre-supplementation mean ± SD = 23.9 ± 11.3 μmol/l), and we also found a rather modest effect of vitamin B6 supplementation on ΔtHcy in our participants with rheumatoid arthritis. In the present study, there was a significant treatment effect of vitamin B6 supplementation (*p* = 0.022) in subjects with an initial ΔtHcy level above 15 μmol/l, suggesting that there may be a threshold effect of pyridoxine on ΔtHcy levels. Treatment with vitamin B6 may only lower ΔtHcy in individuals who start with elevated ΔtHcy levels. Conversely, the disrupted homocysteine metabolism may not be simply due to vitamin B6 inadequacy in these patients as 2 of the 14 subjects in the B6 group with abnormal initial ΔtHcy still had a similarly abnormal response to methionine load after the 30 day vitamin B6 supplementation (ΔtHcy > 30 nmol/l). As elevated ΔtHcy was found to be associated with enhanced disease activity in these patients [5], we suggest that alleviating the disease activity of rheumatoid arthritis with medication may help correct the abnormal methionine load outcomes. Further studies are warranted to study whether suppressing inflammation improves vitamin B6 status. It is also possible that factors other than vitamin B6 status, such as heterozygosity or deficiency of cystathionine β-synthase, may be responsible for the elevated ΔtHcy in these individuals. In this case, vitamin B6 supplementation alone may or may not be sufficient to correct the abnormal outcomes in response to a methionine load test.

We previously demonstrated the potential interfering effect of methotrexate on the methionine load test [2]. In addition, Bruckner *et al*. [25] demonstrated that drug therapy such as corticosteroids may have effects on tryptophan metabolism. We therefore performed block randomization to improve comparability between the B6 and the placebo group and minimize potential confounding effects of medication use. There was no difference in weight, height, age, methotrexate or prednisone dose, duration of disease, number of painful/swollen joints, The Health Assessment Questionnaire (HAQ) disability score, erythrocyte sedimentation rate, or rheumatoid factor between the placebo and B6 group at baseline, indicating that our strategy of randomization of treatment was effective.

## Conclusion

Vitamin B6 supplementation is effective in improving static and functional vitamin B6 status regardless of the etiology of the vitamin B6 deficiency in patients with rheumatoid arthritis with plasma pyridoxal 5'-phosphate below the 25^th ^percentile of the Framingham Heart Cohort Study. All static measurements of vitamin B6 status, including plasma and erythrocyte pyridoxal 5'-phosphate, αEAST, urinary XA excretion in response to a tryptophan load test, and 24 h 4-PA excretion, were significantly improved by the 30 day vitamin B6 treatment. We suggest that vitamin B6 supplementation should be considered in rheumatoid arthritis patients to improve vitamin B6 status, and to reduce the potential adverse consequences of B6 vitamin deficiency. In light of the potential benefits of improving B6 status in patients with rheumatoid arthritis, further studies should be conducted to determine the optimal dose that maximizes the biochemical as well as functional indices reflecting vitamin B6 therapy.

## Abbreviations

4-PA = 4-pyridoxic acid; αEAST = erythrocyte aspartate aminotransferase activity coefficient; CRP = C-reactive protein; ΔtHcy = net homocysteine increase in response to a methionine load test; EAST = erythrocyte aspartate aminotransferase; ESR = erythrocyte sedimentation rate; GCRC = General Clinical Research Center; NEMC = New England Medical Center;  PBMC = peripheral blood mononuclear cells; tHcy = plasma total homocysteine; TNF = tumor necrosis factor; XA = 24 h urinary xanthurenic acid excretion in response to a tryptophan load test.

## Competing interests

The authors declare that they have no competing interests.

## Authors' contributions

All authors made substantive intellectual contributions to the present study. E-PC conceived of the study, acquired partial funding, and carried out the human experiments, including study designs, coordination, biochemical analyses, data acquisition, analysis and interpretation, and drafted the manuscript. JS participated in the design of the study, acquisition of funding, and was involved in revising the manuscript critically for important intellectual content. GED participated in the design of the study and performed the statistical analysis. RR conceived of the study, acquired funding, and performed all clinical assessments in study subjects, and revised the manuscript critically for important intellectual content.

## Figures and Tables

**Table 1:** Description of subjects

	Placebo group (n = 14)	Vitamin B6 group (n = 14)
Age	57.5 (11.0)	53.9 (12.6)
Sex (F:M)	9:5	12:2
Height (cm)	168.4 (10.3)	164.7 (9.1)
Methotrexate (yes/all)	7/14	8/14
Methotrexate dose (mg/week)	7.5 (10.1)	10.2 (11.7)
Prednisone (yes/all)	9/14	11/14
Prednisone dose (mg/week)	3.1 (3.5)	4.3 (4.0)
NSAIDs use (yes/all)	10/14	11/14
Duration of disease (years)	11.6 (8.2)	8.5 (5.6)
Number of painful joints	5.1 (5.1)	7.9 (8.9)
Number of swollen joints	8.9 (8.7)	8.0 (9.5)
The Health Assessment Questionnaire disability score 1–3 scale	1.45 (1.18)	1.17 (0.94)
Erythrocyte sedimentation rate	30.2 (21.4)	36.0 (29.9)
Rheumatoid factor (IU/ml)	87.2 (69.2)	88.8 (82.9)
Albumin (g/dl)	3.8 (0.5)	3.4 (0.4)
Alkaline phosphatase (IU/l)	76.6 (14.5)	73.6 (23.6)
24 h creatinine (mg/dl)	1.02 (0.38)	1.01 (0.42)
C-reactive protein (mg/l)	16.7 (16.2)	8.6 (12.7)

Values represent mean (SD). NSAIDs, non steroidal anti-inflammatory drugs.

**Table 2:** Measurements of vitamin B6 status before and after 30 day treatment

	Placebo group (n = 14)		B6 group (n = 14)		*p* value (baseline)^a^	*p* value (treat)^b^
	Before	After	Before	After		

Plasma PLP (nmol/l)^c^	22.8 (15.4–31.5)	23.6 (15.2–43.0)	27.0 (20.4–30.9)	144.5 (84.5–236.7)	<0.0001	<0.0001
Erythrocyte PLP (nmol/l)	26.0 (20.8–39.4)	41.6 (28.5–53.7)	44.6 (37.5–54.0)	116.4 (65.3–424.7)	0.623	0.002
αEAST	1.88 (1.67–1.99)	1.85 (1.64–1.96)	1.80 (1.68–1.93)	1.33 (1.29–1.40)	0.001	<0.0001
ΔtHcy (μmol/l)^c^	19.2 (15.0–27.5)	17.9 (13.0–25.8)	24.9 (16.4–35.9)	19.0 (15.5–28.7)	<0.0001	0.086
Post-load XA (μmol/24 h)^c^	173 (132–243)	137 (103–354)	183 (30–653)	102 (39–371)	0.001	0.042
4-PA (μg/24 h)	0.7 (0.5–1.2)	0.8 (0.5–170)	0.8 (0.5–2.0)	4.2 (0.8–12.8)	0.338	<0.0001

Data are presented as median (95% CI). ^a^Effects of each baseline (before treatment) value on its post-treatment outcome. ^b^Treatment effects of placebo and vitamin B6 were examined by analysis of covariance after adjusting for baseline value. ^c^Plasma pyridoxal 5'-phosphate (PLP), urinary xanthurenic acid excretion in response to a tryptophan load test (post-load XA), and plasma total homocysteine (tHcy) concentrations were log-transformed to reach normal distribution for statistical analyses. αEAST, erythrocyte aspartate aminotransferase activity coefficient; 4-PA, 24 h 4-pyridoxic acid excretion; ΔtHcy, net homocysteine increase in response to a methionine load test.

**Table 3:** Inflammatory cytokines, C-reactive protein, erythrocyte sedimentation rate, and rheumatoid factor before and after 30 day treatment

	Placebo group (n = 14)		B6 group (n = 14)		*p* value (baseline)^a^	*p* value (treat)^b^
	Before	After	Before	After		

PBMC IL-6 (pg/ml)^c^	490 (289–832)	1,369 (202–1,665)	1,112 (437–1,352)	1,476 (918–1,602)	0.698	0.315
PBMC TNF-α (ng/ml)^d^	224.6 (118.4–361.8)	341.5 (242.6–654.1)	114.1 (319.1–89.2)	178.7 (59.6–391.0)	0.320	0.963
Serum TNF-α (pg/ml)	1.7 (0.7–3.8)	2.1(0.3–5.5)	1.5 (0.9–2.7)	2.0 (0.9–3.6)	0.134	0.166
Serum CRP (mg/l)	13.0 (5.90–27.6)	7.0 (4.4–27.5)	2.0 (0.1–17.2)	3.0 (0.6–14.8)	0.387	<0.0001
ESR	31.0 (19.4–52.6)	32.0 (24.0–49.7)	27.5 (18.8–41.6)	31.0 (22.4–38.9)	0.425	<0.0001
RF (IU/ml)	72.0 (43.3–131.2)	93.8 (37.1–132.5)	76.4 (47.5–130.0)	73.8 (47.3–122.8)	0.697	<0.0001

Data are presented as median (95% CI). ^a^Effects of each baseline (before treatment) value on its post-treatment outcome. ^b^Treatment effects (placebo versus vitamin B6) were examined by analysis of covariance, adjusting for baseline (before) value. ^c^Spontaneous production of IL-6 by peripheral blood mononuclear cells (PBMCs). ^d^Spontaneous production of tumor necrosis factor (TNF)-α by PBMCs. CRP, C-reactive protein; ESR, erythrocyte sedimentation rate; RF, rheumatoid factor.
